# Does inferior alveolar nerve block influence immediate postoperative outcomes in orthognathic surgery? A scoping review

**DOI:** 10.1007/s10006-026-01660-3

**Published:** 2026-11-14

**Authors:** Renan Viana Parente, Yuri Freires Braga, Luis Eduardo Velez Macas, Fábio Wildson Gurgel Costa, Eduardo Frederico Eduardo Maferano

**Affiliations:** 1 Undergraduate Program in Dentistry, Faculty of Dentistry, Federal University of Ceará, Ceará, Brazil; 2 Residency Program in Oral and Maxillofacial Surgery and Traumatology, Dr. José Frota Institute, Ceará, Brazil; 3 School of Dentistry, Universidad Nacional de Loja, Loja, Ecuador; 4 Postgraduate Program in Dentistry, Faculty of Dentistry, Federal University of Ceará, Ceará, Brazil; 5 Department of Dentistry, Faculty of Health Sciences, Zambeze University, Tete, Mozambique

**Keywords:** Nerve block, Inferior alveolar nerve, Postoperative pain, Mandibular osteotomy, Scoping review

## Abstract

**Purpose:**

This scoping review aimed to systematically map and synthesize the available evidence regarding the impact of inferior alveolar nerve (IAN) block on the immediate postoperative outcomes of orthognathic surgery.

**Methods:**

The review was conducted in accordance with the PRISMA-ScR (Preferred Reporting Items for Systematic Reviews and Meta-Analyses Extension for Scoping Reviews) guideline. A comprehensive and systematic search strategy was implemented across MEDLINE (PubMed), EMBASE, CINAHL, PsycINFO, The Cochrane Library, Livivo, LILACS, Web of Science, Scopus, Epistemonikos, and Google Scholar for grey literature. The search employed the descriptors descriptors “Nerve Block,” “Postoperative Pain,” and “Mandibular Osteotomy,” combined with the Boolean operators AND and OR and their respective synonyms. The findings were synthesized narratively.

**Results:**

The search yielded 2,235 records, of which 11 studies fulfilled the eligibility criteria. Substantial heterogeneity was identified regarding the type, dosage, formulation of the anesthetic agents, and timing of IAN block administration. Despite such variations, beneficial effects were consistent across the included studies: 63.6% reported a statistically significant reduction in postoperative pain, 62.5% demonstrated reduced opioid consumption, and 66.7% showed a lower incidence of postoperative nausea and vomiting (PONV). The visual analogue scale (VAS) was the most frequently used instrument for postoperative pain assessment.

**Conclusion:**

The available evidence suggests that the IAN block is associated with reduced postoperative pain, decreased opioid requirements, and a lower incidence of PONV in patients undergoing orthognathic surgery. The heterogeneity observed across anesthetic protocols underscores the need for standardization in future primary studies.

**Supplementary Information:**

The online version contains supplementary material available at https://doi.org/10.1007/s10006-026-01660-3.

## Introduction

Orthognathic surgery aims to correct maxillomandibular deformities that impair essential functions and facial aesthetics, resulting in functional rehabilitation and improved quality of life. These procedures address occlusion, mastication, respiration, speech, temporomandibular joint function and, in selected cases, may contribute to the management of obstructive sleep apnea [1, 2]. Common techniques include Le Fort I osteotomy, genioplasty, and bilateral sagittal split osteotomy (BSSO). Performed under general anesthesia, these interventions involve extensive soft-tissue dissection and bone manipulation, which explains their association with substantial postoperative morbidity, most commonly pain, edema, bleeding, nausea, vomiting, and neurosensory disturbances [3–5].

Among these outcomes, postoperative pain is the most prevalent and clinically relevant complication, representing a major challenge during the immediate recovery period. Pain negatively affects patient comfort, early mobilization, analgesic requirements, and overall postoperative satisfaction, making effective pain control a key component of perioperative care. Standard postoperative management frequently relies on opioid analgesics, which, despite their efficacy, are associated with an increased risk of adverse effects, particularly postoperative nausea and vomiting (PONV) [6]. PONV is among the most distressing complications following general anesthesia, with peak incidence within the first 24 h and a well-established association with perioperative opioid use [2, 7, 8].

In this context, peripheral nerve blocks have been adopted as an adjuvant strategy to improve analgesia, reduce perioperative opioid use, and limit opioid-related adverse effects, including postoperative nausea and vomiting [7]. Ropivacaine is frequently used due to its long duration of action and favorable safety profile, although other agents such as lidocaine and bupivacaine are also commonly reported [5, 6, 8, 9]. Despite this, no consensus exists regarding optimal anesthetic agents or standardized protocols for regional anesthesia in orthognathic surgery. Moreover, the postoperative application of inferior alveolar nerve (IAN) block remains inconsistently investigated [1, 2].

Because BSSO is a common orthognathic procedure, the inferior alveolar nerve plays a key role in postoperative pain modulation [10, 11]. Nevertheless, evidence remains fragmented, particularly regarding protocol standardization, anesthetic selection, and outcome reporting, and no scoping synthesis has systematically mapped these aspects or the patient-reported outcome measures used.

In light of these gaps, a scoping approach is warranted to systematically map, characterize, and summarize the breadth of available evidence. Therefore, this scoping review aims to identify and synthesize the literature on the use of the IAN block in orthognathic surgery, specifically in the context of mandibular BSSO, to evaluate its effectiveness in reducing postoperative pain and opioid-related complications, such as nausea and vomiting. Additionally, this review seeks to describe the anesthetic protocols reported, including block technique, anesthetic agent, volume, use of adjuvants, and timing of administration, and to identify the PROMs applied to assess postoperative impact and quality of life.

## Materials and methods

### Study design

This scoping review was conducted in accordance with the PRISMA-ScR reporting guideline [12] and followed the Joanna Briggs Institute (JBI) methodological framework for scoping reviews, encompassing definition of research questions, identification and selection of studies, data charting, and synthesis of results [13]. The protocol was prospectively registered on the Open Science Framework (OSF) (DOI: https://doi.org/10.17605/OSF.IO/M7G56) before study selection and data extraction, ensuring transparency and reproducibility. According to the Population–Concept–Context (PCC) framework, the population included patients undergoing BSSO, the concept was the use of IAN block, and the context was the immediate postoperative period following orthognathic surgery.

The guiding research questions were defined a priori as follows:

- **Question 1:***What are the reported effects of IAN block on immediate postoperative clinical outcomes following BSSO, including pain intensity, opioid consumption, PONV, intraoperative bleeding, and neurosensory complications?*
- **Question 2:***Which anesthetic protocols for IAN block are described in the literature for patients undergoing BSSO, including block technique, anesthetic agent, dosage or volume, use of adjuvants, and timing of administration?*
- **Question 3:***Which clinical outcomes and outcome measurement methods are reported in the literature for patients undergoing BSSO?*
- **Question 4:***Which PROMs are employed to assess postoperative impact and revovery after BSSO with IAN block, including pain-related instruments (e.g., VAS), oral health-related quality of life measures (e.g., OHIP), and recovery-specific questionnaires?*

### Search strategy

The search strategy was developed to ensure comprehensive, systematic, and reproducible identification of relevant studies, in accordance with PRISMA-ScR recommendations, using a three-step approach proposed by the JBI for scoping reviews. In the first step, an exploratory search was conducted in MEDLINE (PubMed), EMBASE, CINAHL, PsycINFO, The Cochrane Library, LIVIVO, LILACS, Web of Science, Scopus, Epistemonikos, and Google Scholar (grey literature) to identify sentinel and conceptually relevant studies. Controlled vocabulary terms (MeSH and Emtree) and free-text keywords, including “*Nerve Block*,” “*Postoperative Pain*,” and “*Mandibular Osteotomy*,” along with their synonyms, were extracted to refine the final strategy. In the second step, a comprehensive search was performed on October 14, 2025, using the optimized strategy adapted to the syntax of each database, with no restrictions on language or publication date. Full search strategies are provided in Supplementary Material 1. In the third step, the search was updated on December 4, 2025, and complemented by backward citation chaining (snowballing) and citation tracking via Web of Science, to identify additional studies not retrieved through electronic searches.

### Eligibility criteria

Studies were considered eligible if they investigated the use of IAN block in the context of BSSO of the mandible and adopted experimental or quasi-experimental designs, including randomized and non-randomized clinical trials. Analytical observational studies, such as prospective or retrospective cohort studies, case–control studies, and analytical cross-sectional studies, as well as case series, were also included, in line with the broad inclusion strategy recommended for scoping reviews. Studies were excluded if they comprised single case reports, letters to the editor, author replies, editorials, narrative or systematic reviews, book chapters, or experimental studies conducted in animals or in vitro models. No restrictions were applied regarding publication date, language, or geographic location, consistent with the objective of comprehensively mapping the available evidence.

### Screening and selection of studies/sources of evidence

All records retrieved from electronic searches were imported into EndNote Web (Clarivate Analytics, PA, USA) for automatic and manual duplicate removal, after which the deduplicated dataset was uploaded to Rayyan (Qatar Computing Research Institute, Doha, Qatar) to allow secondary duplicate checking and blinded screening. Titles and abstracts were independently screened by two reviewers (RVP and EFEM), blinded to each other’s decisions, according to predefined eligibility criteria, and classified as included, excluded, or potentially eligible. Full texts of potentially eligible studies were then independently assessed, with reasons for exclusion systematically recorded. Disagreements were resolved by consensus, and when necessary, a third reviewer (FWGC) adjudicated the decision. The study selection process is documented in a PRISMA 2020 flow diagram adapted for scoping reviews, in accordance with PRISMA-ScR recommendations.

### Data charting process

Data were independently extracted by two reviewers (RVP and EFEM) using a predefined and standardized form developed a priori. Disagreements were resolved by consensus or, when necessary, by a third reviewer (FWGC). Extracted variables included bibliographic information (authors, year, and geographic location); methodological characteristics (objectives, design, and sample size); population characteristics (sex distribution, age range, and study groups); intervention details (anesthetic technique, type and volume of local anesthetic, and adjuvants); clinical outcomes and assessment instruments; and key findings (main results and authors’ conclusions).

### Data analysis and presentation

The extracted data were synthesized using a descriptive narrative approach to map and summarize the available evidence on preoperative and perioperative anesthetic techniques in patients undergoing BSSO. Consistent with the purpose of a scoping review, no statistical pooling or meta-analysis was performed. Data synthesis prioritized thematic categorization of key variables, enabling identification of patterns, evidence gaps, and heterogeneity across studies.

## Results

### Search strategy and study selection

The systematic search identified 2,235 records. After removal of 653 duplicates using EndNote and Rayyan, 1,582 records were screened by title and abstract, of which 26 articles were retrieved for full-text assessment. Following full-text evaluation, 18 studies were excluded due to unavailable full text (*n* = 10), mandibular fracture populations (*n* = 2), outcomes limited to intraoperative bleeding (*n* = 2), concomitant use of anesthetic block with another substance (*n* = 1), isolated maxillary nerve block (*n* = 1), case report design (*n* = 1), and a previously undetected duplicate (*n* = 1). Excluded studies and reasons are detailed in Supplementary Material 2.

**Fig. 1:**
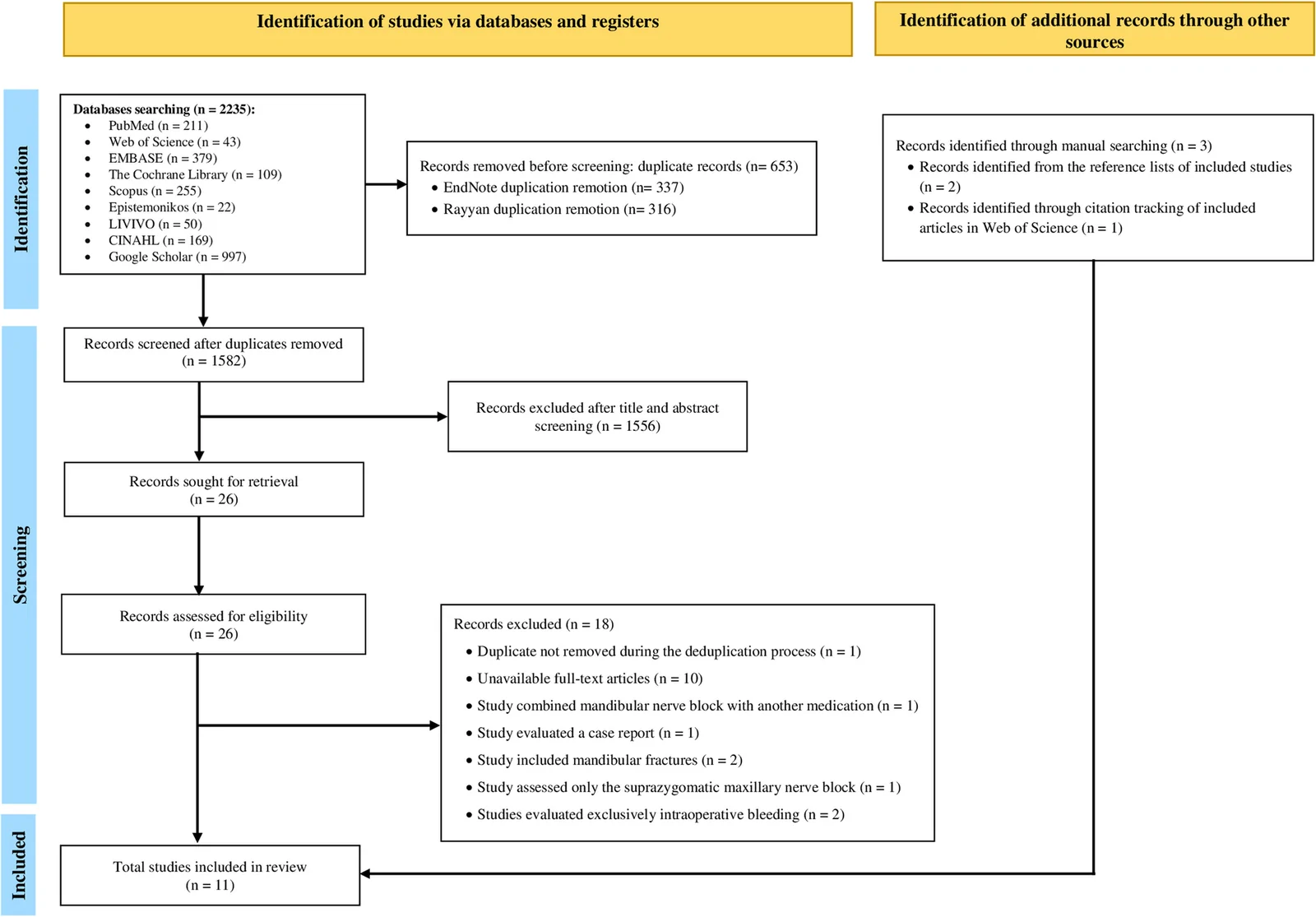
Flow diagram of literature search and selection criteria

To enhance comprehensiveness, manual searches were performed, including backward citation chaining, which identified two additional eligible studies, and citation tracking via Web of Science, which yielded one additional study. In total, 11 studies met the inclusion criteria. The study selection process is depicted in the PRISMA flow diagram (Fig. 1).

### Characteristics of the included studies

**Fig. 2:**
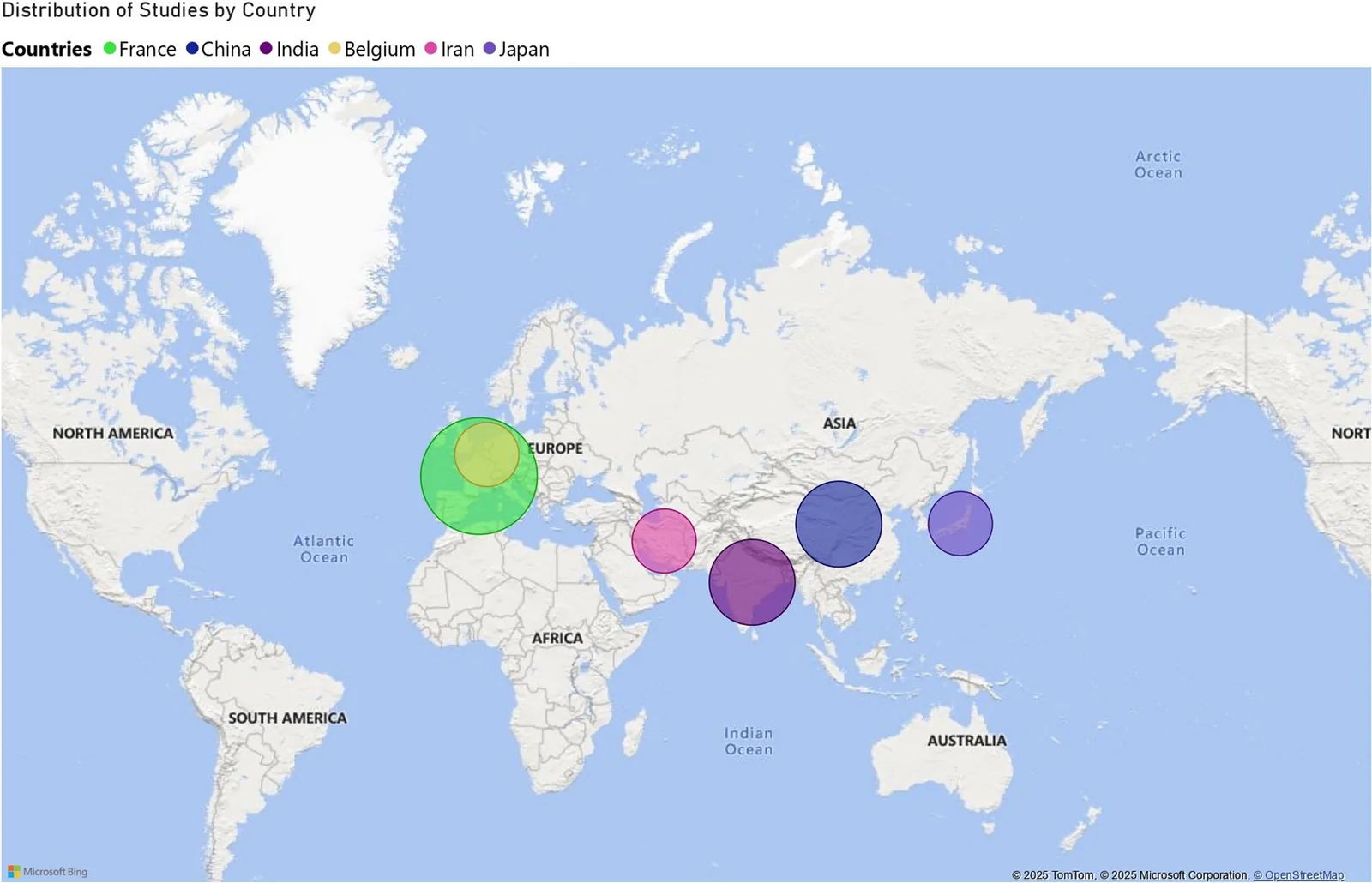
Distribution of studies by country

A total of 11 studies were included [1–9, 14, 15]. All studies evaluated the immediate postoperative period following orthognathic surgery, specifically bilateral mandibular osteotomy, and consistently employed general anesthesia combined with inferior alveolar nerve (IAN) block, with additional maxillary nerve block reported in several studies [1, 4–6, 9, 15]. All studies reported approval by Research Ethics Committees. Seven studies (63.6%) were published within the last five years, indicating growing recent interest. Studies were conducted in Europe and Asia, with France contributing the largest proportion (36.4%), followed by China (18.2%) and India (18.2%), while Belgium, Iran, and Japan each contributed one study. Geographical distribution is shown in Fig. 2. Regarding study design, most studies were prospective (81.8%; *n* = 9), whereas two were retrospective (18.2%). Among prospective studies, 55.6% did not report blinding, 22.2% were single-blind, and 22.2% were double-blind, reflecting methodological heterogeneity. Further methodological details are summarized in Table 1.

**Table 1:** Characteristics of the included studies

Author(s)/ Year	Country (Continent)	Study design	Sample size	Sample characteristics (age, sex distribution)	Analyzed groups	Technique (type of nerve block), and timing of anesthetic administration	Type and dosage of anesthetic used
Chatellier et al. 2012 [7]	France (Europe)	PRCT	30	CG: 25.8 ± 8.4 years; male 37.5%, female 62.5% SG: 24.2 ± 5.4 years; male 50%, female 50%	CG: no block SG: block with ropivacaine	Regional inferior alveolar nerve block. Timing of administration: after general anesthesia.	3 mL of ropivacaine
Espitalier et al. 2011 [3]	France (Europe)	PRCT single-blind	38	CG: 21 ± 11 years; male 42%, female 58% GS: 27 ± 13 years; male 26%, female 74%	CG: no block SG: block with ropivacaine	Mandibular nerve block. Timing of administration: before general anesthesia.	5 mL of ropivacaine (5 mg/mL)
Esquerré et al. 2025 [1]	France (Europe)	PRCT single-blind	43	CG: 23 years SG: 19 years	CG: no block SG: regional block with ropivacaine	Bilateral combined maxillary (V2) and mandibular (V3) nerve blocks. The V2 block was performed via the suprazygomatic approach. The V3 block was ultrasound-guided, with the probe positioned posteriorly relative to the V2 block. The pterygomandibular space was identified between the coronoid process and the condyle with the mouth open. Needle insertion was performed out of plane, through an infrazygomatic approach, near the maxillary artery. Timing of administration: after general anesthesia.	5 mL of ropivacaine (4.75 mg/mL)
Krishnan et al. 2008 [9]	India (Asia)	Prospective case-control clinical trial	20	NR	CG: no block SG: block with bupivacaine	Pre-incisional blocks included infraorbital, posterior superior alveolar, and greater palatine blocks for maxillary procedures; and, for the mandible, mandibular nerve block using the Gow-Gates technique and inferior alveolar nerve block. Timing of administration: after general anesthesia.	2 mL of 0.25% bupivacaine
Oono et al. 2023 [6]	Japan (Asia)	Retrospective study	56	CG: 22.5 years; male 38.2%, female 61.8% SG: 22 years; male 36.4%, female 63.6%	CG: no block SG: block with levobupivacaine	Maxillary and mandibular nerve blocks were performed using anatomical landmark techniques with computed tomography assessment. Timing of administration: after general anesthesia.	0.5% levobupivacaine
Vetter et al. 2020 [2]	France (Europe)	PRCT	51	CG: 22.0 years; male 32.0%, female 68% SG: 24.5 years; male 46.2%, female 53.8%	CG: no block EG: block with ropivacaine	Bilateral inferior alveolar nerve block. Timing of administration: after general anesthesia.	3 mL of ropivacaine
Van Lancker et al. 2003 [8]	Belgium (Europe)	PRCT double-blind	40	CG: 20.3 ± 9.6 years; male 55%, female 45% SG: 19.1 ± 5.8 years; male 45%, female 55%	CG: no block SG: block with lidocaine	Mandibular nerve block. Timing of administration: before general anesthesia.	2 mL of 2% lidocaine
Hosseini-Abrishami et al. 2025 [14]	Iran (Asia)	PRCT	32	CG: 23.3 ± 16.18 years; male 18.7%, female 81.3% SG: 24.5 ± 6.44 years; male 31.2%, female 68.8%	CG: lidocaine with epinephrine SG: ropivacaine	Local anesthetic infiltration at the sites of bilateral sagittal split osteotomy incisions. Timing of administration: not reported.	2% lidocaine with 1:80,000 epinephrine and 0.5% ropivacaine
Shetty et al. 2020 [5]	India (Asia)	PRCT double-blind	110	CG: 20.51 ± 4.740 years; male 43.6%, female 56.4% SG: 21.05 ± 4.703 years; male 45.4%, female 54.6%	CG: no block SG: block with bupivacaine hydrochloride	Extraoral approach as described by Bennett: After identifying the midpoint of the depression along the inferior border of the zygomatic process, a 23-gauge spinal needle marked at 5 cm was inserted perpendicularly to the skin until contacting the lateral pterygoid plate. The needle was then slightly withdrawn and redirected to access the pterygopalatine fossa, where 5 mL of local anesthetic was injected after negative aspiration to block the maxillary branch. For the mandibular division, the needle was redirected posterior to the lateral pterygoid plate and advanced to 5 cm before depositing bupivacaine following negative aspiration. Timing: after general anesthesia.	5 mL of 0.25% bupivacaine
Wang et al. 2021 [4]	China (Asia)	PRCT	40	CG: 24.7 ± 6.2 years; male 50%, female 50% SG: 23.6 ± 3.7 years; male 25%, female 75%	CG: no block SG: block with ropivacaine	The block was performed in the preparation room. Patients were placed in the supine position. The ultrasound probe, covered with a sterile sheath, was positioned beneath the zygomatic arch, superior to the mandibular notch and anterior to the condyle, allowing visualization of the zygomatic bone, lateral pterygoid muscle, lateral pterygoid plate, and maxilla. The needle was inserted in-plane, advancing from lateral to medial and posterior to anterior toward the pterygopalatine fossa. Timing of administration: after induction of general anesthesia.	5 mL of 0.25% ropivacaine
Wu et al. 2024 [15]	China (Asia)	Retrospective cohort study	354	CG: 22.5 ± 4.0 years; male 44.6%, female 55.4% SG: 22.6 ± 4.4 years; male 36.6%, female 63.4%	CG: no block SG: block with ropivacaine	The maxillary nerve was blocked intraorally by injecting into the pterygopalatine canal at the mucosal depression corresponding to the greater palatine foramen. A 25-gauge, 5-cm needle was advanced 2–3 cm, and 2–3 mL of 0.5% ropivacaine with 1:100,000 epinephrine was injected bilaterally after negative aspiration. The inferior alveolar nerve was blocked intraorally at the superior portion of the buccal fat pad (3–4 mm lateral to the midpoint of the pterygomandibular fold and 10 mm above the occlusal plane). A 25-gauge, 5-cm needle was inserted 2–2.5 cm, and 2–3 mL of the same anesthetic solution was injected bilaterally after negative aspiration. Timing: after induction of general anesthesia and before the skin incision.	2–3 mL of 0.5% ropivacaine

All studies reported approval from their respective ethics committees. PRCT, Prospective randomized clinical trial; CG, Control group, SG, study group; NR, Not reported

#### Characteristics and sample size of the included studies

The 11 included studies encompassed a total of 814 patients, with individual sample sizes ranging from 20 to 354 participants. Female predominance was reported in 72.7% of the studies (*n* = 8) [2–7, 14, 15]. One study (9.1%) reported equal sex distribution [8], while two studies (18.2%) did not adequately report sex distribution [1, 9], precluding sex-based analyses. Overall, demographic reporting was heterogeneous. Study characteristics and sample sizes are summarized in Table 1.

#### Characteristics of the study groups

Regarding comparison group structure, most studies (90.9%; *n* = 10) [1–9, 15] used a parallel-group design, comparing IAN block combined with general anesthesia versus general anesthesia alone. One study (9.1%; *n* = 1) [14] compared experimental groups receiving IAN block with different local anesthetic agents, enabling assessment of anesthetic-related effects independent of block performance. Group characteristics are summarized in Table 1.

#### Distribution of the types of nerve blocks assessed

Regarding the type and anatomical extent of nerve blocks, 54.5% of the studies (*n* = 6) [1, 4–6, 9, 15] evaluated combined maxillary and mandibular blocks, whereas 45.5% (*n* = 5) focused exclusively on IAN block [2, 3, 7, 8, 14]. With respect to timing of administration, most studies (72.7%; *n* = 8) reported performing the block after induction of general anesthesia, while two studies (18.2%) applied the block pre-induction, aiming for preemptive analgesia. One study (9.1%) did not report block timing, limiting comparability. Details are summarized in Table 1.

#### Type and volume of anesthetic agents

Regarding local anesthetic agents used for IAN block, ropivacaine was the most frequently reported agent, used in 54.5% of the studies (*n* = 6) [1–4, 7, 15]. Reported volumes included 5 mL (50.0%), 3 mL (33.3%), and 2–3 mL of 0.5% ropivacaine (16.7%). Bupivacaine was employed in 18.2% of studies (*n* = 2), with 2 mL and 5 mL of 0.25% bupivacaine administered, respectively [5, 9]. One study (9.1%; *n* = 1) used 0.5% levobupivacaine [6]. Lidocaine alone was reported in one study (9.1%) [8], and one study [14] used a combined protocol of lidocaine with epinephrine and ropivacaine. Overall, substantial heterogeneity in anesthetic agents and volumes was observed, limiting direct comparability. Anesthetic protocols are detailed in Table 1.

#### Outcomes assessed and measurement instruments

Across the included studies, substantial heterogeneity was observed in outcome definition and prioritization. Only 45.5% (*n* = 5) explicitly differentiated primary and secondary outcomes, whereas 54.5% (*n* = 6) did not, limiting interpretation of outcome hierarchy. Postoperative pain was assessed in all studies, confirming its role as the central outcome, most measured using the visual analogue scale (VAS; 72.7%), while the Numerical Rating Scale (NRS) was used in the remaining studies. Postoperative opioid consumption was evaluated in 72.7% of studies, with objective quantification methods reported in four, descriptive nursing records in one, and unclear assessment methods in two, compromising comparability. Postoperative nausea and vomiting (PONV) were assessed in 54.5% of studies, using either the Apfel score combined with antiemetic use, clinical episode documentation, or unspecified methods. Overall, variability in outcome selection and measurement methods limited cross-study comparability. Intraoperative bleeding was assessed in 45.5% of the studies (*n* = 5) [1, 3, 5, 6, 14], with substantial methodological variability. Measurement approaches ranged from quantitative estimates without methodological detail, to continuous bleeding scales, aspirated volume, and a gravimetric method adjusted for irrigation volume, precluding cross-study comparison. Assessment of other postoperative complications was infrequent. Only one study evaluated paresthesia [9], and one study assessed postoperative headache [4]. A detailed summary of outcomes and assessment methods is provided in Table 2.

**Table 2:** Outcomes measured by the studies included in the review

Author(s)	Measured outcomes
Chatellier et al. 2012 [7]	Primary outcomes: incidence of PONV; resolution of obstructions; postoperative pain; morphine consumption within 24 h; use of antiemetics; patient comfort; and associations with age, sex, medical history, and smoking status.
Espitalier et al. 2011 [3]	Primary outcome: bone bleeding. Secondary outcomes: pain intensity, postoperative morphine consumption, and osteotomy execution time.
Esquerré et al. 2025 [1]	Primary outcome: total morphine consumption within the first 24 h after surgery. Secondary outcomes included cumulative postoperative opioid consumption in the PACU and at 48 h after surgery (i.e., postoperative day 2); highest NRS recorded in the PACU, on day 1, and on day 2 through structured interviews; intraoperative blood loss; consumption of anesthetic agents; duration of anesthesia and surgery; monitoring of adverse effects; and the correlation between anxiety and morphine consumption.
Krishnan et al. 2008 [9]	Primary outcomes: postoperative pain; episodes of nausea, vomiting, and gastric acidity; symptoms of numbness at the surgical site; and any discomfort associated with paresthesia.
Oono et al. 2023 [6]	The primary outcome was postoperative pain. Secondary outcomes included the duration of analgesic requirement (days) and the number of days with pain, as well as intraoperative variables: duration of surgery (min); duration of anesthesia (min); blood loss (mL); volume of 1% lidocaine for infiltration anesthesia (mL); and remifentanil consumption (mg).
Vetter et al. 2020 [2]	Primary outcomes: risk of PONV; postoperative pain; use of antiemetics; and postoperative morphine bolus administration.
Van Lancker et al. 2003 [8]	Primary outcomes: postoperative pain, PONV, and intraoperative sufentanil consumption.
Hosseini-Abrishami et al. 2025 [14]	Primary outcomes: intraoperative blood loss, postoperative pain, and opioid consumption.
Shetty et al. 2020 [5]	The primary outcome was postoperative pain. Secondary outcome variables included postoperative discomfort, analgesic consumption, duration of surgery, and blood loss.
Wang et al. 2021 [4]	The primary outcome was pain intensity. Secondary outcomes included patient satisfaction with pain control during the first 24 postoperative hours; the number of patients who experienced moderate to severe pain at 2, 4, 6, 12, and 24 h after the procedure; the number of patients requiring rescue analgesia; the total volume of intravenous agents administered for anesthetic hypotension control; hemodynamic parameters assessed at six different time points; and the incidence of adverse effects within the first 24 postoperative hours.
Wu et al. 2024 [15]	The primary outcomes were postoperative pain and postoperative vomiting (POV) during the first 24 h.

*PACU* post-anesthesia care unit, *NRS* postoperative pain score, *PONV* postoperative nausea and vomiting

#### Main findings of the included studies

Among the 11 included studies, 90.9% (*n* = 10) [1–9, 15] reported statistically significant differences in at least one assessed outcome. Postoperative pain showed statistically significant between-group differences in 63.6% of the studies evaluating this outcome (*n* = 7) [2–7, 9], with higher pain scores consistently reported in participants who did not receive the regional block. For postoperative opioid consumption, 62.5% of the studies (*n* = 5) [1–3, 5, 8] reported significantly greater opioid use in control groups without regional blockade. Among studies assessing postoperative nausea and vomiting (PONV), 66.7% (*n* = 4) [2, 4, 7, 15] reported a significantly higher incidence in participants who did not receive the nerve block. In contrast, intraoperative bleeding was less consistently affected, with only one study (20%; *n* = 1) [3] reporting a statistically significant difference, characterized by greater bone bleeding in the control group. Headache was evaluated in one study (9.1%; *n* = 1) [4], which reported a higher frequency in the control group. Paresthesia, assessed in a single study [9], showed no statistically significant between-group differences. Finally, one study (9.1%; *n* = 1) [14], in which both groups received mandibular nerve blockade and differed only by the anesthetic agent used, reported no statistically significant differences for any evaluated outcome. Additional details are summarized in Table 3.

**Table 3:** Main findings of the studies included in the review

Author(s)	Main findings
Chatellier et al. 2012 [7]	The anesthetic block significantly reduced the occurrence of PONV within the first 24 h. No significant differences were observed between groups regarding the need for airway deobstruction, use of antiemetics, or number of pain episodes. Mean pain intensity was significantly lower in the block group, whereas opioid consumption and cumulative 24-hour morphine levels remained similar between groups.
Espitalier et al. 2011 [3]	During mandibular osteotomy, the bone bleeding index showed statistically significant differences, being higher in the control group than in the block group. Regarding a completely dry surgical field (bleeding index of zero), statistically significant differences were also observed, occurring less frequently in the control group than in the block group. The duration of mandibular osteotomy was longer in the control group than in the block group, and pain scores—as well as total morphine consumption (including the initial intraoperative bolus)—were higher in the control group compared with the block group, all with statistically significant differences.
Esquerré et al. 2025 [1]	Compared with infiltration anesthesia, there was a statistically significant difference: regional block reduced cumulative morphine consumption on postoperative day 1. However, it did not significantly reduce the incidence of PONV nor the intensity of postoperative pain.
Krishnan et al. 2008 [9]	Patients in the study group reported significantly lower VAS scores than those in the control group at all postoperative time points. Rescue analgesics were administered to fewer patients in the study group compared with the control group, although this difference did not reach statistical significance. Thus, patients in the study group experienced less pain and required fewer analgesics. No patient in either group reported nausea or discomfort related to numbness. In the study group, no patient reported symptoms of gastritis, whereas 60% of patients in the control group did.
Oono et al. 2023 [6]	The intraoperative variables: duration of surgery (min), duration of anesthesia (min), blood loss (mL), volume of 1% lidocaine for infiltration anesthesia (mL), and remifentanil consumption (mg), were higher in the pre-block group. However, the areas under the postoperative pain curves on the visual analogue scale at all assessed time points (6, 12, 18, and 24 h) were lower in the pre-block group, with statistically significant differences. No significant differences were observed between groups regarding the duration of analgesic requirement or the number of days with pain.
Vetter et al. 2020 [2]	The proportion of patients who experienced at least one episode of PONV within the first 24 h after surgery was significantly lower among those who received regional anesthesia. There was no statistically significant evidence of differences between the groups regarding antiemetic consumption. VAS pain scores in the block group were significantly lower, and a significant difference was also observed between groups in median postoperative morphine bolus administration.
Van Lancker et al. 2003 [8]	The control group received significantly more sufentanil during the surgical procedure than the lidocaine block group. No statistically significant differences were observed between groups regarding the number of patients who experienced nausea and vomiting requiring rescue medication in the hospital. Likewise, no statistically significant differences were found in additional pain interventions in the ward. Mean VAS pain scores were low (< 3) in both treatment groups, with no significant differences between them.
Hosseini-Abrishami et al. 2025 [14]	Postoperative opioid consumption was similar between groups. There were no statistically significant differences in the volume of irrigation solution administered or in intraoperative blood loss. No statistically significant differences were observed in postoperative pain levels after orthognathic surgery.
Shetty et al. 2020 [5]	Mean pain scores were lower in the test group than in the control group, showing significant differences at 6, 12, 24, and 48 h after surgery. Reported pain scores were 0 in both groups on the fifth postoperative day. Mean discomfort scores were also lower in the test group compared with the control group, with statistically significant differences at 6, 12, 24, and 48 h. Reported discomfort scores were 0 in both groups on the fifth postoperative day. The mean number of analgesics consumed by patients in the test group was significantly lower than in the control group. A positive correlation was observed between surgery duration and pain intensity at 6, 12, 24, and 48 h.
Wang et al. 2021 [4]	Although no statistically significant difference was observed 2 h after surgery, VAS scores in the block group were lower at subsequent postoperative time points. VAS scores in the block group were significantly lower than those in the non-block group at 6 and 12 h postoperatively, and the number of patients experiencing moderate to severe pain was also significantly reduced at 6 and 12 h in the block group. The 24-hour postoperative VAS score did not differ significantly between groups; however, the number of patients with VAS scores greater than 3 was significantly lower in the block group than in the control group. Patients in the study group reported higher satisfaction with postoperative pain control than those in the control group within 24 h. The incidence of vomiting was significantly higher in the control group than in the block group. The incidence of headache was also significantly higher in the control group. Two cases of gastrointestinal flatulence were reported in the control group and none in the block group, although this difference was not statistically significant. Intraoperative consumption of intravenous agents, including sufentanil, remifentanil, and nicardipine, was higher in the control group.
Wu et al. 2024 [15]	No statistically significant differences were found between groups regarding postoperative pain intensity within the first 24 h, 7.6% in the nerve block group and 10.9% in the PCA group. However, the risk of postoperative vomiting was significantly lower in the nerve block group (38.5% vs. 65.2%).

*VAS* visual analogue scale, *PCA* patient-controlled analgesia

## Discussion

This scoping review systematically mapped and synthesized the available evidence regarding the use of IAN block in orthognathic surgery, with particular emphasis on BSSO. The analysis focused on the breadth and characteristics of the evidence, including clinical effectiveness in postoperative pain control and reduction of opioid-related complications, as well as variations in anesthetic protocols, encompassing block technique, anesthetic agents, administered volumes, and timing of application. In addition, this review identified the PROMs used to assess postoperative impact and recovery. To the best of our knowledge, this is the first scoping review to comprehensively and systematically integrate these dimensions within the context of BSSO, thereby addressing a relevant gap in the literature.

In this review, we observed that most of the included studies [1, 2, 4–6, 14, 15] were published within the past five years, indicating a growing interest in the use of IAN block during sagittal split ramus osteotomy. This increased attention may reflect a search for strategies that enhance postoperative recovery, particularly by improving patient comfort and potentially reducing adverse events associated with opioid use. Furthermore, this trend aligns with the broader movement toward more patient-centered clinical practices [16].

With respect to anesthetic protocols, this scoping review identified substantial heterogeneity in the type, concentration, volume, and formulation of local anesthetics used for IAN block, highlighting the absence of standardized or consensus-based protocols for regional anesthesia in BSSO. Ropivacaine was the most frequently reported agent (54.5%), likely reflecting its favorable pharmacokinetic and pharmacodynamic profile, including prolonged duration of action and lower cardiovascular and central nervous system toxicity compared with other long-acting anesthetics [1–5, 7, 15]. Bupivacaine, used in 18.2% of studies, remains a commonly adopted alternative, largely due to its comparable duration of analgesia [5, 9].

Regarding the timing of anesthetic administration, 72.7% of the included studies [1, 2, 4–7, 9, 15] reported performing the IAN block after induction of general anesthesia. In contrast, only two studies [3, 8] described administering the block prior to induction, whereas one study [14] did not specify the timing. These findings highlight substantial heterogeneity in the temporal application of the regional nerve block, which complicates direct comparisons across studies.

The studies included in this review primarily assessed four outcomes: postoperative pain, opioid consumption, PONV, and intraoperative bleeding. Two additional secondary outcomes, incidence of paresthesia and occurrence of headache, were also examined. Among the 11 included articles [1–9, 14, 15] all investigated postoperative pain, and among these, 63.6% [2–7, 9] identified statistically significant differences between comparison groups. In all of these studies, pain scores were higher among participants who did not receive the regional block compared with those who underwent IAN block. These findings reinforce the clinical advantage of performing the regional mandibular nerve block in sagittal split ramus osteotomy, as postoperative pain exerts a direct influence on patient recovery as well as on other postoperative outcomes. Lower pain intensity tends to reduce opioid requirements, which in turn decreases the likelihood of adverse effects associated with this drug class, such as nausea and vomiting.

Furthermore, most studies employed the VAS to assess postoperative pain. This predominant use is justified by its simplicity, sensitivity to small variations in pain intensity, and high reproducibility, which make it a standardized and widely applicable method in clinical research. Because it requires minimal instruction and allows continuous pain recording, the VAS facilitates comparisons between groups and across studies, reinforcing its recurrent adoption in the literature [17, 18].

Postoperative opioid consumption was evaluated in 72.7% of the included studies [1–5, 7, 8, 14]. Among these, more than half [1, 2, 4, 14] quantified opioid use objectively, whereas one study [7] relied on nursing records and two [3, 5] did not clearly describe the assessment method. Most studies analyzing this outcome (62.5%) [1–3, 5, 8] reported significantly higher opioid consumption among patients who did not receive the regional block, reinforcing its positive impact on postoperative analgesia.

This reduction in opioid requirements is particularly meaningful because these medications are typically used as adjunctive analgesic strategies. Thus, patients receiving the regional block tend to report lower pain scores and therefore require smaller opioid doses compared with those who do not undergo the technique [1–3, 5, 8]. The consistency of this association strengthens the evidence that regional anesthesia contributes to more efficient and potentially safer postoperative pain management.

Among the studies investigating PONV, 66.7% (*n* = 4) [2, 4, 7, 15] reported a higher incidence of these events in the groups that did not receive the regional block. This finding is consistent with the reduced opioid consumption observed in previous studies [1–3, 5, 8], given that opioids are well-established risk factors for PONV [19, 20]. Considering that PONV is a multifactorial and highly discomforting complication, often perceived by patients as more distressing than postoperative pain itself, the reduction in these episodes represents an important clinical benefit [19].

Regarding the measurement strategies, two studies [2, 7] used the Apfel score, which estimates the risk of PONV based on independent predictive variables, whereas two others relied on medical record reviews [4, 15]. Although methodologically distinct, both approaches are adequate for assessing differences between groups without compromising the interpretation of the available evidence. This methodological convergence strengthens the robustness of the findings linking regional nerve block to a lower incidence of PONV.

With respect to intraoperative bleeding, only one study [3] identified a statistically significant difference, reporting greater bone bleeding in the control group compared with the group receiving the regional block. This finding is plausible considering that the local anesthetic was administered in combination with a vasoconstrictor, whose pharmacological action reduces vascular caliber and tissue blood flow, potentially resulting in decreased blood loss during the procedure.

From a physiological perspective, this finding contrasts with the intrinsic effects of local anesthetics, which, when administered alone, typically induce vasodilation and therefore are not expected to reduce intraoperative bleeding [21]. Accordingly, the difference reported by Espitalier et al. [3], is more plausibly attributed to the pharmacological action of the anesthetic–vasoconstrictor combination rather than to the IAN block itself. In the remaining studies [1, 5, 6, 14], no significant between-group differences were observed, even when vasoconstrictors were used, suggesting that the hemodynamic effects of this combination may be influenced by operative variables, vasoconstrictor dosage, or methodological factors.

Finally, regarding paresthesia, only one included study evaluated this outcome [9] and did not identify statistically significant differences between groups. This finding is consistent with current evidence, as postoperative paresthesia is predominantly related to mechanical trauma (e.g., nerve stretching or laceration), thermal injury from rotary instruments, or chemical irritation, rather than to the pharmacological effects of local anesthetic agents themselves [22, 23].

This scoping review has limitations. The lack of standardization in anesthetic agents, dosages, and formulations limits direct comparability across studies, as differences in pharmacokinetic and pharmacodynamic profiles may affect both analgesic efficacy and adverse outcomes. In addition, the predominance of prospective studies without blinding increases the risk of selection and outcome assessment bias. Another important limitation is the absence of validated, procedure-specific instruments to assess postoperative quality of life in orthognathic surgery, which restricts the evaluation of patient-centered outcomes. Despite these limitations, this review presents notable strengths. It systematically maps the available evidence on local anesthetic agents used for inferior alveolar nerve block in sagittal split ramus osteotomy and, to the best of our knowledge, represents the first scoping review to compare outcomes between patients managed with and without IAN block in combination with general anesthesia. These findings provide a structured foundation for future research and may inform evidence-based perioperative pain management strategies.

## Conclusion

This scoping review indicates that IAN block is consistently associated with improved immediate postoperative pain control and reduced opioid consumption following sagittal split ramus osteotomy, with a potential reduction in postoperative nausea and vomiting. Ropivacaine was the most frequently reported anesthetic agent. However, substantial heterogeneity in anesthetic protocols, timing of block administration, and outcome assessment limits comparability across studies. Future research should focus on standardized anesthetic protocols and validated, PROMs to strengthen the evidence base and support more consistent evaluation of patient-centered outcomes.

## Supplementary Information

Below is the link to the electronic supplementary material.

Supplementary Material 1 (PDF 158 KB)

## Data Availability

No datasets were generated or analysed during the current study.
