# Supplementary material for: Does inferior alveolar nerve block influence immediate postoperative outcomes in orthognathic surgery? A scoping review
Source: Oral Maxillofac Surg. 2026 Nov 14;30(1):176. doi: 10.1007/s10006-026-01660-3 (PMC13768378; doi:10.1007/s10006-026-01660-3)
Supplement: Supplementary file 1 [file 10006_2026_1660_MOESM1_ESM.pdf]

Supplementary Material Table S1 Search Strategy

Database	Search Strategy
**PubMed**	((("Nerve Block"[Mesh] OR "Nerve Block*" OR "Local Anesthetics" OR "Inferior Alveolar Nerve Block" OR "Mandibular Nerve Block" OR "Alveolar Nerve Block" OR "Regional Anesthesia" OR "Regional Analgesia" OR "Anesthetic Technique*")) AND (("Pain, Postoperative"[Mesh] OR "Postoperative Pain" OR "Post-operative Pain" OR "Pain after surgery" OR "Post-surgical Pain" OR "Pain, Acute Postoperative" OR "Pain, Chronic Postoperative" OR "Persistent Postsurgical Pain" OR "Postoperative Analgesia"))) AND (("Mandibular Osteotomy"[Mesh] OR "Mandibular Osteotomy" OR "Bilateral Sagittal Split Osteotomy" OR "BSSO" OR "Orthognathic Surgery"[Mesh] OR "Orthognathic Surgery" OR "Mandibular Surgery" OR "Jaw Surgery" OR "Oral Surgical Procedures"[Mesh] OR "Mandible"[Mesh]))
**Embase**	('nerve block'/exp OR 'nerve block*':ti,ab OR 'local anesthesia'/exp OR 'local anesthesia':ti,ab OR 'local anesthetic agent'/exp OR 'local anesthetics':ti,ab OR 'inferior alveolar nerve block':ti,ab OR 'mandibular nerve block':ti,ab OR 'alveolar nerve block':ti,ab OR 'regional anesthesia'/exp OR 'regional anesthesia':ti,ab OR 'regional analgesia':ti,ab OR 'anesthetic technique*':ti,ab) AND ('postoperative pain'/exp OR 'postoperative pain':ti,ab OR 'post-operative pain':ti,ab OR 'pain after surgery':ti,ab OR 'post-surgical pain':ti,ab OR 'acute postoperative pain'/exp OR 'acute postoperative pain':ti,ab OR 'chronic postoperative pain':ti,ab OR 'persistent postsurgical pain':ti,ab OR 'postoperative analgesia'/exp OR 'postoperative analgesia':ti,ab) AND ('mandibular osteotomy'/exp OR 'mandibular osteotomy':ti,ab OR 'bilateral sagittal split osteotomy'/exp OR 'bilateral sagittal split osteotomy':ti,ab OR 'bsso':ti,ab OR 'orthognathic surgery'/exp OR 'orthognathic surgery':ti,ab OR 'mandibular surgery':ti,ab OR 'jaw surgery':ti,ab OR 'oral surgical procedure' OR 'oral surgical procedures':ti,ab OR 'mandible'/exp OR 'mandible':ti,ab)
**Scopus**	(TITLE-ABS-KEY ("nerve block*" OR "local anesthesia" OR "local anesthetics" OR "inferior alveolar nerve block" OR "mandibular nerve block" OR "alveolar nerve block" OR "regional anesthesia" OR "regional analgesia" OR "anesthetic technique*") AND TITLE-ABS-KEY ("postoperative pain" OR "post-operative pain" OR "pain after surgery" OR "post-surgical pain" OR "acute postoperative pain" OR "chronic postoperative pain" OR "persistent postsurgical pain" OR "postoperative analgesia") AND TITLE-ABS-KEY ("mandibular osteotomy" OR "bilateral sagittal split osteotomy" OR "BSSO" OR "orthognathic surgery" OR "mandibular surgery" OR "jaw surgery" OR "oral surgical procedures" OR "mandible"))
**Web of science**	("nerve block*" OR "local anesthetics" OR "inferior alveolar nerve block" OR "mandibular nerve block" OR "alveolar nerve block" OR "regional anesthesia" OR "regional analgesia" OR "anesthetic technique*") (All Fields) and ("postoperative pain" OR "post-operative pain" OR "pain after surgery" OR "post-surgical pain" OR "acute postoperative pain" OR "chronic postoperative pain" OR "persistent postsurgical pain" OR "postoperative analgesia") (All Fields) and ("mandibular osteotomy" OR "bilateral sagittal split osteotomy" OR "basso" OR "orthognathic surgery" OR "mandibular surgery" OR "jaw surgery" OR "oral surgical procedures" OR "mandible") (All Fields)
**CINAHL**	((MH "Nerve Block+") OR (MH "Anesthesia, Local+") OR (MH "Anesthetics, Local+") OR "nerve block*"

**(EBSCO)**	OR "local anesthetics" OR "inferior alveolar nerve block" OR "mandibular nerve block" OR "alveolar nerve block" OR "regional anesthesia" OR "regional analgesia" OR "anesthetic technique*") AND ((MH "Pain, Postoperative+") OR (MH "Pain Management+") OR "postoperative pain" OR "post-operative pain" OR "pain after surgery" OR "post-surgical pain" OR "acute postoperative pain" OR "chronic postoperative pain" OR "persistent postsurgical pain" OR "postoperative analgesia") AND ((MH "Osteotomy, Sagittal Split+") OR (MH "Mandibular Surgery+") OR (MH "Orthognathic Surgery+") OR (MH "Mandible+") OR "mandibular osteotomy" OR "bilateral sagittal split osteotomy" OR "BSSO" OR "orthognathic surgery" OR "mandibular surgery" OR "jaw surgery" OR "oral surgical procedures" OR "mandible")
**LIVIVO**	((("nerve block*" OR "local anesthetic*" OR "inferior alveolar nerve block" OR "mandibular nerve block" OR "alveolar nerve block" OR "regional anesthesia" OR "regional analgesia" OR "anesthetic technique*")) AND ("postoperative pain" OR "post-operative pain" OR "pain after surgery" OR "post-surgical pain" OR "acute postoperative pain" OR "chronic postoperative pain" OR "persistent postsurgical pain" OR "postoperative analgesia")) AND ("mandibular osteotomy" OR "bilateral sagittal split osteotomy" OR "BSSO" OR "orthognathic surgery" OR "mandibular surgery" OR "jaw surgery" OR "oral surgical procedure*" OR "mandible")
**The Cochrane Library**	("nerve block*" OR "local anesthetics" OR "inferior alveolar nerve block" OR "mandibular nerve block" OR "alveolar nerve block" OR "regional anesthesia" OR "regional analgesia" OR "anesthetic technique*") in Title Abstract Keyword AND ("postoperative pain" OR "post-operative pain" OR "pain after surgery" OR "post-surgical pain" OR "acute postoperative pain" OR "chronic postoperative pain" OR "persistent postsurgical pain" OR "postoperative analgesia") in Title Abstract Keyword AND ("mandibular osteotomy" OR "bilateral sagittal split osteotomy" OR "BSSO" OR "orthognathic surgery" OR "mandibular surgery" OR "jaw surgery" OR "oral surgical procedures" OR "mandible") in Title Abstract Keyword - (Word variations have been searched)
**Epistemonikos**	(title:((title:(("nerve block" OR "local anesthetics" OR "inferior alveolar nerve block" OR "mandibular nerve block" OR "regional anesthesia" OR "regional analgesia" OR "anesthetic technique")) OR abstract:(("nerve block" OR "local anesthetics" OR "inferior alveolar nerve block" OR "mandibular nerve block" OR "regional anesthesia" OR "regional analgesia" OR "anesthetic technique"))) AND (title:(("postoperative pain" OR "post-surgical pain" OR "pain after surgery" OR "postoperative analgesia")) OR abstract:(("postoperative pain" OR "post-surgical pain" OR "pain after surgery" OR "postoperative analgesia"))) AND (title:(("orthognathic surgery" OR "mandibular osteotomy" OR "bilateral sagittal split osteotomy" OR "BSSO" OR "mandibular surgery" OR "jaw surgery" OR "oral surgical procedures" OR "mandible")) OR abstract:(("orthognathic surgery" OR "mandibular osteotomy" OR "bilateral sagittal split osteotomy" OR "BSSO" OR "mandibular surgery" OR "jaw surgery" OR "oral surgical procedures" OR "mandible")))) OR abstract:((title:(("nerve block" OR "local anesthetics" OR "inferior alveolar nerve block" OR "mandibular nerve block" OR "regional anesthesia" OR "regional analgesia" OR "anesthetic technique")) OR abstract:(("nerve block" OR "local anesthetics" OR "inferior alveolar nerve block" OR "mandibular nerve block" OR "regional anesthesia" OR "regional analgesia" OR "anesthetic technique"))) AND (title:(("postoperative pain" OR "post-surgical pain" OR "pain after surgery" OR "postoperative analgesia")) OR abstract:(("postoperative pain" OR "post-surgical

	pain" OR "pain after surgery" OR "postoperative analgesia"))) AND (title:(("orthognathic surgery" OR "mandibular osteotomy" OR "bilateral sagittal split osteotomy" OR "BSSO" OR "mandibular surgery" OR "jaw surgery" OR "oral surgical procedures" OR "mandible")) OR abstract:(("orthognathic surgery" OR "mandibular osteotomy" OR "bilateral sagittal split osteotomy" OR "BSSO" OR "mandibular surgery" OR "jaw surgery" OR "oral surgical procedures" OR "mandible")))))
**Google Scholar (grey literature)**	("nerve block" OR "local anesthetic" OR "mandibular nerve block" OR "regional anesthesia" OR "anesthetic technique") AND ("postoperative pain" OR "post-surgical pain" OR "pain after surgery" OR "postoperative analgesia") AND ("orthognathic surgery" OR "mandibular osteotomy" OR "bilateral sagittal split osteotomy" OR "BSSO" OR "jaw surgery" OR "mandibular surgery")

Supplementary Material Table S2 List of excluded articles and corresponding reasons for exclusion

Author/Year	Reason for exclusion	Reference
Chatellier et al., 2012	Excluded because it was an unremoved duplicate	Chatellier A, Dugué AE, Cafourier C, Maksud B, Compère JF, Bénateau H. Bloc du nerf alvéolaire inférieur par ropivacaïne: effets sur les nausées et vomissements en postopératoires (NVPO) des ostéotomies mandibulaires. Rev Stomatol Chir Maxillofac. 2012;113(6):417-22. doi:10.1016/j.stomax.2012.10.003.
Epistallier et al., 2007	The article evaluated intraoperative bleeding exclusively	Espitalier, Fabien & Dubost, A & Ehrmann, Stephan & Laffon, Marc & Fusciardi, J. (2007). Ropivacaïn mandibular nerve block decreases bleeding during sagittal mandibular osteotomy: 8AP5-6. European Journal of Anaesthesiology - EUR J ANAESTH. 24. 10.1097/00003643-200706001-00354.
Epistallier et al., 2008	The article evaluated intraoperative bleeding exclusively	E. Fabien; F. Espitalier; A. Dubost; M. Laffon; D. Goga; J. Fusciardi. (2008). O.381 Mandibular nerve block reduces osteotomy's bleeding. 36(supp-S1). doi:10.1016/s1010-5182(08)71505-1
Zhang et al., 2020	The article analyzed the association between nerve block administration and intravenous analgesia with parecoxib sodium	Zhang D, Cao Y, Lin J. Clinical observation of inferior alveolar nerve block combined with intravenous analgesia with parecoxib sodium after orthognathic mandibular surgery. J Prev Treat Stomatol Dis. 2020;28(5):303-6. doi:10.12016/j.issn.2096-1456.2020.05.006.
Inacio et al., 2016	Case report	Inacio R., Saura C., Amaro S., Teresa E., Alexandre G., Ribeiro J., Reis E., Vilaca M., Rodrigues I., Faisco A. (2016). Maxillary and mandibular nerve block for orthognathic surgery postoperative analgesia-case report, 35th Annual European Society of Regional Anaesthesia and Pain Therapy Congress, ESRA 2016. Regional Anesthesia and Pain Medicine. http://dx.doi.org.ez11.periodicos.capes.gov.br/10.1097/AAP.0000000000000469
Bertuit et al., 2020	Excluded because it addressed mandibular fractures	Bertuit M., Rapido F., Ly H., Vannucci C., Ridolfo J., Molinari N., De Boutray M., Galmiche S., Dadure C., Perrigault P.-F., Capdevila X., Chanques G. (2021). Bilateral mandibular block improves pain relief and morphine consumption in mandibular osteotomies: A prospective, randomized, double-blind, placebo-controlled clinical trial. Regional Anesthesia and Pain Medicine, 46(4), 322-327. http://dx.doi.org.ez11.periodicos.capes.gov.br/10.1136/rapm-2020-102417
Green et al., 2024	Article unavailable	Lidocaine Versus Bupivacaine in Orthognathic Surgery: A Randomized Controlled Trial (2024), Identifier: NCT06450028. https://clinicaltrials.gov/study/NCT06450028
*2023	Excluded because it addressed mandibular fractures	Effect of Combined Maxillary and Mandibular Nerve Block on Orthognathic Surgery Outcomes (2023), Identifier: NCT05925465. https://clinicaltrials.gov/study/NCT05925465
*2019	The article focused exclusively on the suprazygomatic maxillary nerve block	Effects of Ultrasound-guided Bilateral Suprazygomatic Maxillary Nerve Block on Postoperative Pain After Elective Bimaxillary Osteotomy in Adult Patients (2019), Identifier: NCT03913429. https://clinicaltrials.gov/study/NCT03913429
*2022	Article unavailable	Analgesic Efficacy of Echoguided Maxillary and Mandibular Nerve Blocks in Planned Maxillomandibular Osteotomy Surgery (2022), Identifier: NCT05351151.

		https://clinicaltrials.gov/study/NCT05351151
*2011	Article unavailable	Intra-operative Trigeminal Nerve Block For Post-operative Pain Control After Orthognathic Surgery (2011), Identifier: NCT01418183. https://clinicaltrials.gov/study/NCT01418183
*2015	Article unavailable	Effects of the Bilateral Mandibular Nerve Block (V3) in Mandibular Osteotomy: a Prospective, Randomized, Double-blind Versus Placebo Study (2015), Identifier: NCT02618993. https://clinicaltrials.gov/study/NCT02618993
Ballabriga et al., 2019	Article unavailable	Does it Worth to Reinforce With Additional Anesthesia to Improve Postoperative Course After Orthognathic Surgery? (2019), Identifier: NCT03974035. https://clinicaltrials.gov/study/NCT03974035
Kumita et al., 2023	Article unavailable	Kumita S, Chaki T, Sawada A, Yamakage M. Effect of ultrasound-guided maxillary and inferior alveolar nerve block in two-jaw plastic surgery: a single-blind randomised controlled trial in two centres. *Reg Anesth Pain Med.* 2023;48(Suppl 1):A156. doi:10.1136/rapm-2023-009956.
Henry et al., 2020	Article unavailable	Henry A., Whitehead S., Mehra P. (2020). Decreasing Opioid Usage during Hospitalization in Patients Undergoing Orthognathic Surgery, AAOMS 2020. Journal of Oral and Maxillofacial Surgery. http://dx.doi.org/10.1016/j.joms.2020.07.039
Fahmy et al., 2019	Article unavailable	Fahmy MD, Cho RA, Brooks SR, Campbell J, Gotcher JE. Reduction of postoperative narcotic usage following mandibular orthognathic surgeries: is Exparel an effective agent? J Oral Maxillofac Surg. 2019;77(9):e65-e66. doi:10.1016/j.joms.2019.06.087.
Cho et al., 2019	Article unavailable	Cho R., Brooks S., Fahmy M.D., Campbell J. (2019). Reduction of postoperative narcotic usage following mandibular orthognathic surgery: efficacy of liposomal bupivicaine examine, ICOMS 2019: 24th International Conference on Oral and Maxillofacial Surgery. International Journal of Oral and Maxillofacial Surgery. http://dx.doi.org/10.1016/j.ijom.2019.03.762
*2024	Article unavailable	Cochrane Central Register of Controlled Trials. Comparison of the effect of different volumes ropivacaine on ultrasound-guided trigeminal nerve block in patients undergoing bimaxillary orthognathic surgery: a single-center, randomized, parallel-controlled, double-blind, non-inferiority trial [Internet]. 2024. Available from: https://www.cochranelibrary.com/central/doi/10.1002/central/CN-02673989/full

* No author(s) listed.
